# Interplay Between VCAM-1 and PGE2 Levels and Autism Spectrum Disorder Severity in Children—A Preliminary Single-Center Analysis

**DOI:** 10.3390/children12111488

**Published:** 2025-11-03

**Authors:** Irakli Natroshvili, Tatia Gakharia, Sophia Bakhtadze, Tamar Natsvlishvili, Nana Khachapuridze

**Affiliations:** Givi Zhvania Pediatric University Clinic, Tbilisi State Medical University, 33 Vazha-Pshavela Avenue, 0186 Tbilisi, Georgia

**Keywords:** autism spectrum disorder, autism severity, cytokines, biomarkers

## Abstract

**Highlights:**

**What are the main findings?**

- VCAM-1 is higher in the moderate and severe ASD behavior group than in mild cases and declines with age; PGE_2_ did not differ between severity and age groups.
- Elevated VCAM-1 is linked to greater behavioral severity in ASD.

**What is the implication of the main finding?**

- VCAM-1 could be potential biomarker of ASD severity.
- Autism Spectrum Disorders (ASDs) may involve neuroimmune dysregulation.

**Abstract:**

Background: The clinical heterogeneity of autism spectrum disorders (ASDs) results from dynamic interactions between genetic susceptibility and environmental exposures. Autism is increasingly recognized as involving neuroimmune dysregulation, which may contribute to ASD severity. Several studies indicate that ASD patients exhibit increased levels of VCAM-1, suggesting endothelial dysfunction and enhanced leukocyte infiltration into the brain, which may have adverse bearing on synaptic plasticity, axon growth, and repulsion. Similarly, elevated PGE2 drives microglial activation and excitotoxicity. The present study examines possible links between VCAM-1 and PGE2 levels and ASD severity. Methods: VCAM-1 and PGE_2_ concentrations were measured in children with ASD aged 2–6 years and analyzed for age effects and correlations with behavioral severity. Participants were grouped as mild, moderate, or severe based on Autism Diagnostic Observation Schedule-2 (ADOS-2) scores. Results: VCAM-1 levels were subnormal in 39.3% (*n* = 24), and PGE_2_ levels were above normal in 32.8% (*n* = 20). Mean VCAM-1 levels decreased significantly with age (F(4, 56) =2.98, *p* = 0.026) and also, were higher in moderate (U = 36.00, Z = −3.96, *p* < 0.001) and severe (U = 155.50, Z =−2.70, *p* = 0.007) ASD groups, with mean ranks rising from 14.46 (mild) to 41.13 (severe). PGE_2_ did not differ between severity and age groups (*p* > 0.05). VCAM-1 correlated moderately with ADOS-2 scores (rho = 0.577, *p* < 0.001), whereas PGE2 did not (rho = 0.108, *p* = 0.406), suggesting higher VCAM-1 is linked to increased behavioral severity in ASD. Conclusions: Inflammation-related biomarkers could be reflecting a heterogeneous set of neuroimmune mechanisms underlying ASD, which may drive behavioral outcomes.

## 1. Introduction

Autism spectrum disorder (ASD) is a neuropsychiatric disorder presenting with difficulties in social interaction and communication. It could be manifested with restricted interests and behaviors. ASD is a heterogeneous condition with different clinical presentation and severity of signs and symptoms, as well as with various levels of support needed [1].

In recent decennia, the awareness of ASD as a cause of developmental disability increased considerably [2]. For example, the prevalence of ASD diagnosis increased from 1 in 150 in 2000 to 1 in 36 in 2020 in the USA, a continuous multicenter network surveillance of autism and developmental disabilities [3]. ASD manifests primarily in the first three years of life and is characterized by repetitive behavior or interests, deficits in verbal and non-verbal language, and difficulties interacting with others [4]. The most valid test for diagnosing of ASD still remains the Autism Diagnostic Observation Schedule (ADOS). This is a 45-min observation by a professional or clinician to diagnose autism from the age of 12 months to adulthood [5].

The etiology of ASD is considered to be a combination of genetic and environmental factors [3]. Almost 40 years ago, alterations in the immune system were found in ASD patients. Several pieces of evidence suggest that in the brains of patients diagnosed with ASD, there is an ongoing process of neuroinflammation with activation of astroglia and microglia. This may even be the result of maternal infection during pregnancy. Studies have shown that maternal infection increases a child’s risk of developing ASD [6,7].

In ASD patients, abnormal cytokine levels have been observed in the peripheral blood [3,8], as well as reduced cytotoxic activity and elevated levels of proinflammatory cytokines such as interleukin 1 (IL-1) and tumor necrosis factor (TNF-1) [9,10].

In contrast, Singh and colleagues could not find a difference of levels of interleukine-1 (IL-1) in children with ASD and controls [11]. It was confirmed that IL-1-induced activation of the c-Jun N-terminal kinases (JNK) pathway in neurons is mediated by X-linked interleukin-1 receptor accessory protein-like 1 (IL1RAPL1). The gene producing this member of the interleukine-1 receptor family is situated on the X chromosome (Il1RAPL1) and known to be associated with X linked non-syndromic developmental delay [12].

Opposite to this, Singh et al., report the elevated concentration of Interleukin-2 (IL-2) in children with ASD in comparison to matched controls [11]. The activity of natural killer cells (NK cells) is significantly lower in 45% of children with autism spectrum disorders, in accordance with Vojdani and colleagues. Additionally, IL-2 prominently induced NK cell activity in a subgroup of patients with ASD who had extremely low levels of NK cell activity [13]. In plasma, Singh et al. could not detect a difference in levels of Interleukin-6 (IL-6 between an ASD patient group and controls) [14]. In contrast, Wei and colleagues found increased levels of IL-6 in the cerebellum of children with ASD [15]. Xu and colleagues demonstrated that IL-6 overexpression in granule cells of the mouse with experimental ASD causes impairment of adhesion and migration on granule cells but had a non-significant impact on the formation of dendritic spines or granule cell apoptosis [16].

In order to gain access to the brain, leucocytes need to pass the blood–brain barrier (BBB) [5]. Facilitation of the process of white cell migration and modulation of the permeability of the BBB to immune cells could be provided by adhesion molecules [17]. Adhesion molecules—platelet endothelial adhesion molecule-1 (PECAM-1), intercellular adhesion molecule (ICAM-1), vascular adhesion molecule (VCAM-1), P-selectin, and L-selectin are present in BBB membranes in bound form and can also be found in circulating plasma under normal non-inflammatory conditions. Neuroinflammatory diseases may, however, lead to atypical concentrations of these adhesion molecules in the blood [18]. Onore and colleagues found that the median plasma level of soluble PCAM-1 (sPECAM-1) as well as of P-selectin were reduced by 25% in 2–4 year-old ASD children compared to controls. The plasma levels of L-selectin, VCAM-1, or soluble ICAM-1 were the same in the ASD group and controls. Using the Repetitive Behavior Scale, a negative correlation was found between the sPECAM-1 level and the scores on the scale, which means that when the sPECAM-1 level decreases, repetitive behaviors become more severe in ASD children. Significant correlation between P-selectin and clinical behavior in the same study was not found [5].

Kameno and colleagues report that the serum levels of VCAM-1 and sPECAM-1 were significantly lower in 5–17 year-old children than in controls [18]. However, they could not find a correlation between the levels of sPECAM-1 and VCAM-1 and clinical variables, including results on the Autism Diagnostic Interview-revised (ADI-R), age, weight, and height. In contrast, in 2–4-year-old children with ASD, sPECAM-1 but not VCAM-1 was significantly lower than in controls [5]. In adults with an ASD diagnosis, a similar situation was found as in the 5–17-year-olds, with the difference that VCAM-1 levels only showed a trend towards lower levels in ASD patients [19,20]. They conclude that VCAM-1 only plays a limited role in the pathophysiology of ASD with a more important role for PECAM-1 [18].

In addition, the crucial role in the mechanism of ASD could be played by alterations in cytokine levels and also prostaglandins. The catalytic action of cyclooxygenase (COX) enzymes—cyclooxygenase-1 (COX-1) and cyclooxygenase-2 (COX-2)—synthesizes the latter bioactive lipid compounds from arachidonic acid. They contribute significantly to a range of physiological functions, most notably, the control of inflammatory pathways and the immune system’s responses. Prostaglandin E2 (PGE2) can play a role in the modulation of synaptic function and neuronal activity. PGE2 mediates its actions via four separate G-protein-coupled receptors (EP1–EP4), each of which shows region-specific expression in the brain and participates in controlling neurotransmitter release, synaptic remodeling, and neuroinflammatory responses [21]. Aberrant prostaglandin signaling has been increasingly recognized as relevant to neuropsychiatric disorders, including ASD, as they are involved in brain development and functioning [22]. Dysregulated prostaglandin–COX pathways may interfere with these key mechanisms and thereby play a role in the cognitive and behavioral impairments characteristic of ASD. The level of PGE2 is increased in ASD, suggesting a role in the pathogenesis of this disease [23]. An elevated PGE2-driven chronic inflammatory state may interfere with typical neuronal maturation and synaptic activity, thereby contributing to ASD pathophysiology. Despite gaps in understanding the specific involvement of PGE2 and VCAM-1 in ASD, available evidence points compellingly to their relevance in the disease process. At present, however, the clinical value as a potential easily obtainable biomarker for ASD severity has not been properly addressed.

The present study examines possible links between VCAM-1 and PGE2 levels and the behavioral phenotypes associated with ASD.

## 2. Materials and Methods

### 2.1. Patients

This is a single center cross-sectional study of 2–6-year-old children who visited the outpatient clinic of the Givi Zhvania University Clinic of Pediatrics, Tbilisi, Georgia, during the period between March 2023 and May 2025 with a suspicion of an ASD developmental disorder.

The study excluded participants with recent infections or ongoing inflammation, autoimmune disease, patients with developmental delays with confirmed metabolic disease or genetic alterations, epilepsy, obsessive-compulsive disorder, affective disorder, and any other neurological and mental health diseases. Comorbid psychiatric illnesses were excluded using the Structured Clinical Interview for DSMIV (SCID). There were no dietary supplements taken by patients with diagnosis of ASD, all of them were drug naïve.

A select cohort was administered a special autism assessment questionnaire for parents—the AUTISM DIAGNOSTIC INTERVIEW-REVISED (ADI-R) [19]—confirming a suspicion of autism by parents and neurologists. After an ADI, all patients with suspected ASD, were evaluated by Autism Diagnostic Observation Schedule™, Second Edition (ADOS-2) (Phrase speech, not fluent) modules 1–4 [20]—to accurately determine the severity of ASD by a certified neuropsychiatrist.

Due to the age and verbal ability influencing the raw ADOS-2 modules’ total score, the study group of children diagnosed with ASD was split into three groups according to ADOS-2 converted algorithm comparison scores. With this score, symptoms can be compared across modules and over time based on a scale of 1–10 (calibrated severity score). The groups were classified according to ASD severity as follows: Group 1—Mild severity of ASD-related behaviors, scores 1–3; Group 2—Moderate severity of ASD-related behaviors, scores 4–5; Group 3—Severe ASD-related behaviors, scores 6–10 [19]; higher scores indicate more severe autism spectrum-related behaviors.

### 2.2. Laboratory Investigations

A blood sample was collected from all selected patients who had ADOS-2 confirmed ASD and who had not had an infection of any etiology (including SARS-CoV-2) within the two months prior to enrollment. To exclude fever-related or other inflammatory processes, CBC and C-reactive protein levels were assessed at the time of sampling. Participants with evidence of an inflammatory process based on complete blood count or C-reactive protein results were excluded from the study to minimize the influence of acute ongoing inflammation another genesis.

Collected venous blood samples (400 μL for VCAM-1, 100 μL for PGE2) were centrifuged at 3000 rpm for 15 min and forwarded to the laboratory for further cytokine analysis.

Serum VCAM-1 and PGE2 quantified using ELISA; for measuring VCAM-1, ELISA kits were used from Invitrogen (Thermo Fisher Scientific, Waltham, MA, USA)—The KHT0601 (1. KHT0601—Human VCAM-1 ELISA Kit (Assay Range: 0.59–75 ng/mL, Analytical Sensitivity: <0.5 ng/mL, Assay Type: Sandwich ELISA), and for PGE2- KHL1701 (KHL1701—Human Prostaglandin E2 (PGE2) ELISA Kit, Assay Range: 31.3–4000 pg/mL, Sensitivity: 13.4 pg/mL, Assay Type: Competitive ELISA). Normal ranges based on local laboratory references; for VCAM-1 −410–1160 ng/mL; for PGE2- 25–960 pg/mL.

### 2.3. Statistics

Statistical analysis was performed using IBM SPSS Statistics v24.0. Group comparisons were conducted for VCAM-1 and PGE2 concentrations in sera, and cytokine levels are presented means with confidence intervals and mean ranks. A one-way ANOVA was performed to examine the effect of age on cytokine levels. For comparisons across severity groups, (Mild, Moderate, Severe ASD-related behaviors) where normality was not satisfied, we used the Kruskal–Wallis test followed for Post-Hoc Analysis by the pairwise Mann–Whitney U tests and Dunn’s test with Bonferroni correction. Spearman’s rank correlation was used to explore the association between cytokine levels and severity scores.

### 2.4. Ethics

The study was carried out according to the rules of the Helsinki declaration; detailed written rationale for the study was presented to parents or legal guardians. They were informed that the study would be freely given and would not provide them with medical benefits, and parental or legal guardian consent was obtained; they volunteered their consent to processing the data.

## 3. Results

Study included 61 children aged 2 to 6 years diagnosed with ASD. They were divided into three groups based on ADOS2 calibrated scores: Group 1 with mild ASD-related behaviors (*n* = 13), Group 2 with moderate ASD-related behaviors (*n* = 22), and Group 3 with severe autism spectrum-related behaviors (*n* = 26). The gender ratio among them was boys, 67.2% and girls, 32.7%, distributed into subgroups: in Group 1, mild ASD-related behaviors, Girls *n* = 8 and Boys *n* = 5; in Group 2, Moderate ASD-related behaviors, Girls *n* = 7 and Boys *n* = 15; in Group 3, Severe ASD-related behaviors, Girls *n* = 5 and Boys *n* = 19.

VCAM-1 levels were subnormal in 39.3% (*n* = 24), and 60.7% (*n* = 37) of participants had results within normal ranges based on provided local lab reference (410–1160 ng/mL). PGE2 levels showed almost similar distribution 67.2% (*n* = 41) within normal ranges (25–960 pg/mL); 32.8% (*n* = 20) of them had results above >1000 pg/mL.

The analysis showed a statistically significant difference among age groups for VCAM-1 (F(4, 56) = 2.98, *p* = 0.026). Mean VCAM-1 levels decreased with age significantly (F(4, 56) = 2.98, *p* = 0.026): highest in the 2–3 year children (956.2 ± 38.9 ng/mL) and lowest in the oldest participants, 5–6 year old (763.1 ± 29.9 ng/mL). Age does not appear to influence circulating PGE_2_ levels F(4, 56) = 0.204, *p* = 0.935. See Table 1.

**Figure 1:**
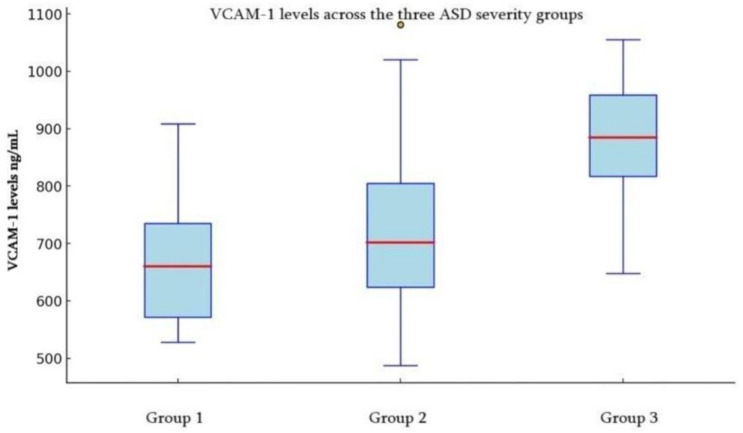
The box plot of VCAM-1 levels (ng/mL) for the three ASD severity groups: Group 1: Mild ASD-related behaviors, Group 2 Moderate ASD-related behaviors, Group 3 Severe ASD-related behaviors on ADOS-2. °—outliers. Red line in the figure—Mean VCAM-1.

By comparing each cytokine separately in groups, analysis of VCAM1 levels across groups revealed a progressive increase from Group 1 (M = 615.08 ng/mL, 95% CI [502.88, 727.29]) to Group 2 (M = 789.05 ng/mL, 95% CI [719.12, 858.99]) and Group 3 (M = 912.41, 95% CI [857.94 ng/mL, 966.89]), with decreasing variability across groups (Figure 1, Table 2).

**Figure 2:**
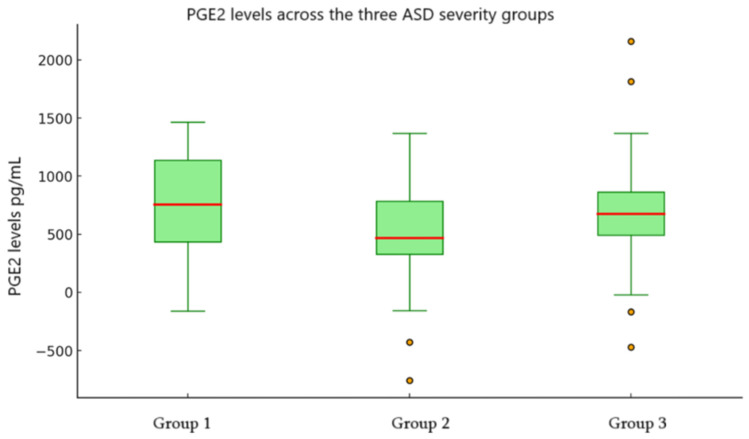
Box plot of PGE2 levels (pg/mL) across the three severity groups. Group 1: Mild ASD-related behaviors. Group 2: Moderate ASD-related behaviors. Group 3: Severe ASD-related behaviors. Red line in the figure -Mean PGE2; °—outliers.

The result of comparing PGE2 levels in different groups: similarly, PGE2 levels showed an increasing trend; increased from Group 1 (M = 543.90 pg/mL, 95% CI [187.53, 900.27]) to Group 2 (M = 601.48 pg/mL, 95% CI [371.63, 831.34]) and Group 3 (M = 681.62 pg/mL, 95% CI [439.25, 924.00]); although variability remained high across all groups (Figure 2, Table 3).

Mann–Whitney U tests were conducted to compare VCAM1 and PGE2 levels between boys and girls. In comparison, boys showed higher mean ranks than girls for both VCAM-1 (mean rank: 33.80 vs. 25.25) and PGE2 (mean rank: 33.90 vs. 25.05). However, neither difference reached statistical significance using the Mann–Whitney U test: VCAM-1 (U = 295.00, Z = −1.77, *p* = 0.077) and PGE2 (U = 291.00, Z = −1.83, *p* = 0.067). These results indicate trends toward higher VCAM-1 and PGE2 levels in boys, but the differences were not statistically significant in this sample (*p* = 0.077 and *p* = 0.067, respectively).

**Figure 3:**
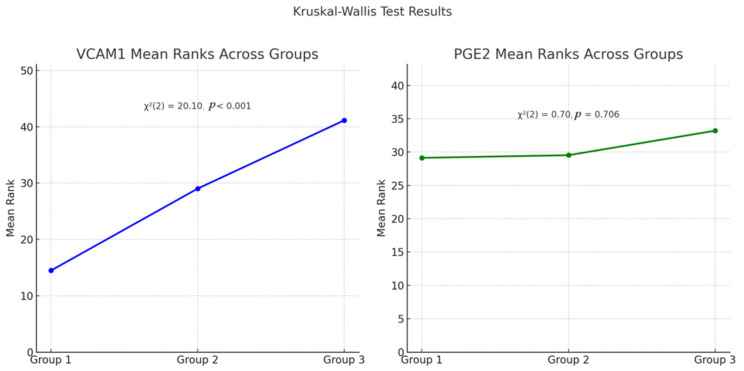
Rank plot. VCAM-1 and PGE2 mean ranks across study groups. Group 1: Mild ASD-related behaviors. Group 2: Moderate ASD-related behaviors. Group 3: Severe ASD-related behaviors.

The Kruskal–Wallis test revealed a statistically significant difference between VCAM-1 levels across the three groups (χ^2^(2) = 20.10, *p* < 0.001). The mean ranks of VCAM-1 increased progressively from Group 1 (mean rank = 14.46) to Group 3 (mean rank = 41.13), indicating higher VCAM-1 concentrations in the moderate and high severity groups. In contrast, PGE2 levels did not differ significantly between groups (χ^2^(2) = 0.70, *p* = 0.706), with mean ranks remaining relatively stable across groups (29.12, 29.52, and 33.19, respectively). See Figure 3 below.

The differences in VCAM-1 levels between the three severity groups were analysed using the Mann–Whitney U test. The analysis revealed a statistically significant difference in VCAM-1 levels (v U = 61.00, Z = −2.80, *p* = 0.005), with Group 2 exhibiting significantly higher VCAM-1 values (mean rank = 21.73) compared to Group 1 (mean rank = 11.69).

Similarly, the results indicated a significant difference in VCAM-1 concentrations (U = 36.00, Z = −3.96, *p* < 0.001), with Group 3 showing a higher mean rank (25.12) than Group 1 (9.77) and there was a significant difference in VCAM-1 levels between Group 3 compared to group 2 (U = 155.5, Z = −2.70, *p* = 0.007), which had higher VCAM-1 concentrations, as indicated by a higher mean rank (29.52) compared to Group 2 (18.57).

To account for multiple comparisons, a Bonferroni correction was applied, adjusting the significance threshold to α = 0.05/3 = 0.017. In the first analysis (*n* = 35), the differences between group 1 and group 2 (U = 61.00, Z = −2.80, *p* = 0.005) remained significant after Bonferroni correction (*p* < 0.017). In the second analysis (*n* = 39), comparison between group 1 and group 3 showed a highly significant difference (U = 36.00, Z = −3.96, *p* < 0.001), also surviving Bonferroni correction. In the third analysis (*n* = 48), between group 2 and group 3, the difference (U = 155.50, Z = −2.70, *p* = 0.007) likewise remained statistically significant after correction. Taken together, the analyses indicate that VCAM1 levels are significantly higher in Group 3 compared to both Group 1 and Group 2, with differences ranging from medium to large effect sizes (see Table 4).

**Figure 4:**
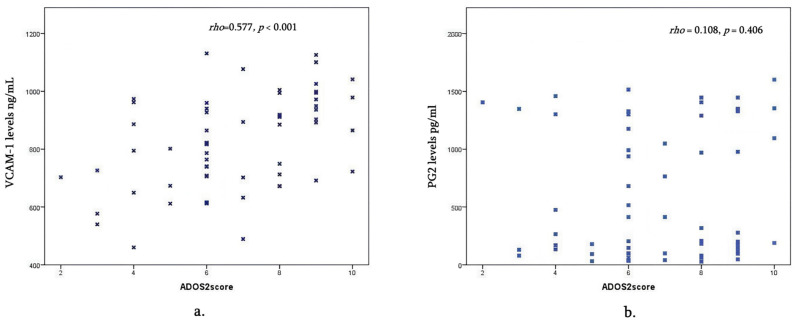
Correlation between cytokines and ADOS2 scores; rho = Spearman’s rho coefficient: (**a**) VCAM1 and ADOS2 scores; (**b**) PGE2 and ADOS2 scores.

Spearman’s correlation analysis was used to examine the relationships between VCAM-1, PGE2, and ADOS2 scores. VCAM-1 had a significant moderate positive correlation with ADOS2 scores (rho = 0.577, *p* < 0.001), indicating that higher VCAM-1 levels are associated with higher ADOS2 scores. In contrast, PGE2 showed no significant correlation with either VCAM-1 (rho = 0.164, *p* = 0.206) or ADOS2 scores (rho = 0.108, *p* = 0.406). See Figure 4.

## 4. Discussion

Autism arises from a multifactorial etiology, where genetic predisposition interacts with neurodevelopmental, immune, and environmental factors, leading to the diverse manifestations of ASD [24,25]. Autism is increasingly understood as a neurodevelopmental condition with an immune component. Numerous studies show that individuals with ASD have altered immune profiles, where prenatal inflammation increases risk and persistent neuroimmune dysregulation after birth may contribute to symptom severity. Altered levels of peripheral immune markers in serum and cerebrospinal fluid are consistently reported [26,27]. Some biomarkers correlate with symptom severity: higher pro-inflammatory cytokines are linked to more severe social and communication difficulties [28].

We investigated the association between serum proinflammatory cytokine levels VCAM-1 and PGE2 and the severity of behavioral phenotypes of ASD based on ADOS2 calibrated scores in 2–6-year-old children diagnosed with ASD. About 61% of participants had VCAM-1 levels in the normal range, and roughly 39% were below it. PGE2 showed a similar pattern, with approximately one-third above the normal cutoff and the rest within range, based on the provided local lab references.

It is important to notice that there is no “single” normal range for VCAM-1 for all children, as levels depend on age, health, and specific conditions. There are several studies where the age-related normal ranges for VCAM-1 were identified with a range of 359.6–822.0 ng/mL for the examined age interval from 6 to 15 year-old healthy children. VCAM-1 levels can vary, with some indicating no significant age dependence in certain age groups [29]. Based on our results, mean VCAM-1 levels decreased significantly with age, highest in the 2–3 y children (956.2 ± 38.9 ng/mL), and lowest in the oldest participants, 5–6 years old (763.1 ± 29.9 ng/mL), which matches the natural decline.

Comparing VCAM-1 levels showed that levels differed significantly between the ASD three severity groups; according to our findings, the level of VCAM-1 is almost normal in Group 1 (mild cases) of ASD (mean 615.08 ng/mL) compared to Group 2 (moderate cases) (mean-789.05 ng/mL) and Group 3 (severe cases) (mean-912.41 ng/mL), with mean ranks rising from 14.46 (mild) to 41.13 (severe), suggesting meaningful differences between severe, moderate, and mild severity groups. VCAM-1 levels were significantly higher in moderate (U = 36.00, Z = −3.96, *p* < 0.001) and severe (U = 155.50, Z = −2.70, *p* = 0.007) ASD groups.

Our data is not consistent with other previous findings. According to Kameno et al., subjects with high-functioning ASD had significantly decreased levels of VCAM-1 compared to controls [18]. When authors examined the correlations between serum levels of VCAM-1 and clinical variables among ASD subjects, they did not find significant clinical variables including age and other parameters. Kameno and colleagues postulate the increased levels of other cytokines like IL-1β, IL-1RA, IL-5, IL-8, IL-12 (p70), IL-13, Il-17, and GRO-α, which correlate with the severity of ASD. Authors concluded that unlike sPECAM-1, which could be considered an important biomarker in the pathogenesis of ASD, VCAM-1 has no significant impact on the clinical course of ASD [18]. The same findings were presented by Bryn and colleagues, who speculated that the cytokine profile of children diagnosed with ASD, regardless of the subdiagnosis, does not differ from healthy controls. Yet, subgroup analysis shows significant variation in IL-8 and IL-10 concentrations, indicating that divergent mechanisms could underlie the different ASD subtypes [30].

Napolioni and colleagues present the same results, that there is no increase in cytokine levels in a set of 40 inflammatory parameters in the peripheral blood of children with ASD compared to matched controls but this study did not aim to assess the level of VCAM-1 [31].

These results were confirmed by another author. Ashwood et al. also proved that there is no difference between plasma levels of cytokines of children with ASD and healthy controls [32].

Although all of these data postulate that cytokines have no role in the mechanism of ASD, the implication of an immunological activation in the prenatal period is very important [33].

According to Gomez-Fernandez, although there is no difference in cytokine levels among ASD children and healthy controls, there could be various parameters for different types of ASD children [34]. In the ASD group with non-neurodevelopmental regression, lower plasma levels of adhesion molecules were detected compared to the levels in the ASD group with neurodevelopmental regression and the control group. It is important to notice that children with ASD have non-neurodevelopmental regression. The authors have observed a higher level of nerve growth factor (NGF) compared to typically developing children. The plasma level of VCAM-1 was studied in high-functioning adults with ASD, where a decreased level of VCAM-1 was found [20,35]. The similar findings were demonstrated by the Kameno group, where the level of VCAM-1 was decreased in male children with ASD with an average age of 11 y [18].

Thus, our study is the first among all research where the level of VCAM-1 seemed to be increased in ASD children, unlike those where a normal or even reduced level of VCAM-1 was found. A reduced level was also proved in adults with ASD, which could be explained by the fact that even in normal adults, the normal range for VCAM-1 is lower (349–991 ng/mL) compared to normal children.

In a large Norwegian birth cohort, higher VCAM-1 measured in newborn cord blood and maternal mid-gestation plasma was among the markers linked with greater later ASD risk (part of an inflammatory/angiogenic signature; sex-stratified effects noted) [36]. This indicates that elevated or dysregulated VCAM-1 early on may signal increased ASD risk later in childhood. However, those studies generally did not measure severity directly, since diagnosis occurs years later.

Our study confirms that children aged 2–6 years showed increased levels of VCAM-1 in patients with moderate and severe ASD-related behaviors. According to our study, VCAM1 and ADOS-2 score had a moderate positive correlation (rho = 0.56), which was statistically significant; thus, possibly higher VCAM1 levels are associated with higher ADOS-2 scores.

From the literature, in some cohorts, lower VCAM-1 correlated with higher severity of autism symptoms—especially social and communication difficulties. This suggests that reduced endothelial/immune adhesion signaling might be linked to worse behavioral profiles. Striking results were obtained from Columbia University and Norwegian Autism Birth Cohort (ABC) utilizing maternal mid-gestation and newborn cord blood samples [36]. Authors found that levels of the following markers were elevated in child cord blood of ASD boys: VCAM-1, EGF, TNFα, Serpin E1, and IL7, as well as in maternal mild gestational plasma. In girls, 13 analytes showed increased concentrations—the five previously noted as elevated in ASD boys’ cord blood, plus CCL5 (RANTES), IL-1RA, IL-13, IL-2, IL-5, FGF-b, and EGF. This evidence is consistent with our study results, where an elevated level of VCAM-1 was found in the plasma of ASD children.

Findings suggest stage-specific biology: prenatal/early-life elevations in VCAM-1 may mark inflammatory or angiogenic processes linked to ASD risk, whereas in diagnosed children/adolescents, peripheral reductions in adhesion molecules (including VCAM-1) are observed [36]. Though typically observed, links to symptom severity are not consistently significant. These patterns point toward stage-dependent processes in ASD’s neuroimmune biology.

In our study, boys showed higher average VCAM1 and PGE2 levels than girls. The confidence intervals for VCAM1 partially overlapped, indicating some variability but notable differences in central tendency between genders. However, a trend toward higher VCAM1 in boys was observed. Boys had a higher mean rank than girls; this difference approached but did not reach statistical significance.

In this study, the second cytokine examined, PGE2, showed an overall increase in mean levels. However, the considerable variability and wide confidence intervals potentially reflect underlying heterogeneity in the sample and substantial data dispersion. Age does not appear to influence circulating PGE_2_ levels. In contrast to all studies conducted before, our study found no meaningful association between PGE2 levels and ADOS2 scores (rho = 0.108, *p* = 0.406).

Elevated PGE2 has been reported in some children with ASD, correlating with markers of immune activation. Increased PGE2 may enhance microglial activation, contributing to synaptic pruning alterations or abnormal neural connectivity [37]. Maternal inflammation or infection during pregnancy can increase fetal PGE2, potentially affecting neuronal migration and synapse formation, which potentially influences neurodevelopmental trajectories linked to ASD risk [38].

El-Ansary and colleagues demonstrated that COX-2 and PEG2 receptors exhibit higher specificity and sensitivity based on ROC curve analysis, highlighting their potential as predictive biomarkers in ASD patients [39].

Growing evidence suggests that prostaglandins and COX could have an actual role in the pathogenesis of ASD based on the theory that chronic neuroinflammation is observed in individuals with ASD [40]. Increased levels of PGE2 have been reported in the serum of ASD individuals, speculating its role in the disorder’s pathogenesis. Increased expression of COX-2 was observed in the blood of patients with ASD [41]. The chronic inflammatory state may disrupt normal neuronal development and synaptic function contributing to ASD mechanism. Moreover, experimental animal studies indicate that COX-2 deficiency induces behavioral abnormalities analogous to those observed in ASD, providing additional support for its involvement in the disorder’s pathogenesis [42].

Recently, Kamal and colleagues confirmed the results of previous colleagues, postulating that prostaglandins and COX enzymes play a significant role in the pathogenesis of ASD. Dysregulation of these biochemical compounds is associated with ASD symptoms. It is important to emphasize that prostaglandins and COX enzymes modulate Wnt signaling pathways and regulate dendritic arborization and cerebellar function, which are crucial to neurodevelopment. Authors conclude that elevated PGE2 and COX-2 show promise as biomarkers for ASD [43].

Higher PGE2 levels in plasma or CSF have been tentatively linked to greater severity of social and communication deficits [44].

Qasem and colleagues proved the previous results in terms of elevated levels of PGE2 in ASD individuals [45], but they detected a very challenging finding that, although ASD patients have remarkably higher levels of the measured parameters compared to neurotypical controls, the exception is the subtype of PGE2, like PGE-EP2 receptors, that showed an opposite trend. Although no correlation was observed between the measured parameter and the severity of social or cognitive deficits, PGE2, COX-2, and mPGES-1 showed a strong association with impairments in sensory processing.

Thus, the relationship between PGE2 levels and ASD symptoms is not straightforward. This variability may be due to differences in study design, sample populations, age, and measurement techniques. Such findings highlight the complexity and variability of inflammatory markers in ASD.

In summary, while there is evidence suggesting altered PGE2 signaling in ASD, the direction and magnitude of these changes are not consistent across studies. Further research is needed to clarify the role of PGE2 in ASD and to determine whether it could serve as a reliable biomarker or therapeutic target.

Accordingly, while interpreting the apparent contrast, it is likely that VCAM1 and PGE2 remain as yet research biomarkers in ASD that are used to probe immune-endothelial and neurovascular pathways for therapeutic implications.

Our study is the first which shows increased levels of VCAM-1 in ASD subgroups and, moreover, revealed a correlation between the level of VCAM-1 and ASD severity, which is contrary to all other similar studies performed before. This finding could help to make a tentative ASD diagnosis, especially in milder cases where diagnosis is difficult to set. Thus, it needs further studies to consider VCAM-1 as a possible biomarker for ASD. Although we presume to consider VCAM-1 as a potential biomarker, it could not be used in milder cases where the level of VCAM-1 was almost equal to the normal range.

This study has several limitations. First, the sample size is small, and the age of the study group is limited, which may limit the statistical power and generalizability of the findings. We had no control group, and it was difficult for us to identify the precise normative data in this pediatric age group. Results vary according to age, sample type, and comorbidities. The results should be interpreted cautiously for proper considerations and a clear understanding of ASD pathogenesis in general; studying a larger cohort with older children is important. Future studies with larger, multicenter cohorts and longitudinal designs are warranted to validate.

## 5. Conclusions

It is important to note that ASD is the result of dynamic interactions between genetic susceptibility and environmental exposures that disrupt critical neurodevelopmental and immune pathways. Inflammation is not the sole cause of ASD but may act as a contributing factor. Understanding the role of inflammation-related biomarkers offers insights into the neuroimmune mechanisms underlying ASD and reflecting a heterogeneous set of biological mechanisms converging on shared behavioral and cognitive outcomes. It may inform future therapeutic approaches targeting inflammatory pathways, which could lead to the identification of subgroups of children with autism who may benefit from targeted treatments.

## Figures and Tables

**Table 1:** VCAM-1 (ng/mL) and PGE2 levels (pg/mL) in children by age groups (Mean ± Standard error of the mean).

Age Group (Years)	VCAM-1 (Mean ± SE)	PGE_2_ (Mean ± SE)
2–3	956.2 ± 38.9	683.2 ± 226.1
3–4	873.0 ± 57.6	577.5 ± 174.7
4–5	834.7 ± 42.2	609.3 ± 154.7
5–6	763.1 ± 29.9	643.3 ± 106.4

**Table 2:** VCAM-1 levels (ng/mL) across the three study groups: Group 1, Mild ASD-related behaviors; Group 2, Moderate ASD-related behaviors; Group 3, Severe ASD-related behaviors. *n* = number of patients. SE—Standard Error, CI—Confidence Intervals, SD—Standard Deviation.

Group	*n*	Mean VCAM-1	SE	95% CI (Lower–Upper)	SD	Skewness
Group 1	13	615.08	51.50	502.88–727.29	185.68	0.88
Group 2	22	789.05	33.63	719.12–858.99	157.74	0.37
Group 3	26	912.41	26.45	857.94–966.89	134.87	−0.50

**Table 3:** PGE2 levels (pg/mL) across the three study groups: Group 1, Mild ASD-related behaviors; Group 2, Moderate ASD-related behaviors; Group 3, Severe ASD-related behaviors on ADOS-2. SE—Standard Error, CI—Confidence Intervals, SD—Standard Deviation.

Group	Mean PGE2	SE	95% CI (Lower–Upper)	SD	Skewness
Group 1	543.90	163.56	187.53–900.27	589.73	0.85
Group 2	601.48	110.53	371.63–831.34	518.42	0.38
Group 3	681.62	117.68	439.25–924.00	600.07	0.26

**Table 4:** Multiple Comparisons of VCAM-1 levels between study groups. Group 1: Mild ASD-related behaviors. Group 2: Moderate ASD-related behaviors. Group 3: Severe ASD-related behaviors. N = number of participants.

Comparison	*n*	U Value	Z Score	Mean Rank	*p*-Value
Group 1 vs. Group 2	35	61.00	−2.80	11.69 vs. 21.73	0.005
Group 1 vs. Group 3	39	36.00	−3.96	9.77 vs. 25.12	<0.001
Group 2 vs. Group 3	48	155.50	−2.70	18.57 vs. 29.52	0.007

## Data Availability

The data presented in this study are available on request from Tbilisi State Medical University in compliance with the organization’s internal standards and ethical reasons. Patient-related data will be shared on request to maintain anonymization of the individual patients.

## Abbreviations

The following abbreviations are used in this manuscript:

ASD	Autism Spectrum Disorder
VCAM-1	Vascular Adhesion Molecule
PGE 2	Prostaglandin 2
ADOS 2	Autism Diagnostic Observation Schedule-2
TNF-α	Tumor Necrosis Factor-α
IL	Interleukin
JNK	c-Jun N-terminal kinases
IL1RAPL1	Interleukin-1 (IL-1) Receptor Accessory Protein-like 1
NK	Natural Killer
CNS	Central Nervous System
BBB	Blood Brain Barrier
PECAM-1	Platelet Endothelial Adhesion Molecule-1
ICAM-1	Intercellular Adhesion Molecule-1
sVCAM-1	Serum Vascular Adhesion Molecule-1
sPECAM-1	Soluble Platelet Endothelial Adhesion Molecule-1
COX	Cyclooxygenase
COX-1	Cyclooxygenase-1
SCID	Structured Clinical Interview for DSMIV
ADI-R	Autism Diagnostic Interview-Revised
